# Epigallocatechin-3-Gallate as a Potential Therapeutic Drug for TTR-Related Amyloidosis: “In Vivo” Evidence from FAP Mice Models

**DOI:** 10.1371/journal.pone.0029933

**Published:** 2012-01-10

**Authors:** Nelson Ferreira, Maria João Saraiva, Maria Rosário Almeida

**Affiliations:** 1 Grupo de Neurobiologia Molecular, IBMC – Instituto de Biologia Molecular e Celular, Universidade do Porto, Porto, Portugal; 2 Departamento de Biologia Molecular, ICBAS – Instituto de Ciências Biomédicas Abel Salazar, Universidade do Porto, Porto, Portugal; Federal University of Rio de Janeiro, Brazil

## Abstract

**Background:**

Familial amyloidotic polyneuropathy (FAP) is a neurodegenerative disease caused by the extracellular deposition of mutant transthyretin (TTR), with special involvement of the peripheral nervous system (PNS). Currently, hepatic transplantation is considered the most efficient therapy to halt the progression of clinical symptoms in FAP since more than 95% of TTR is produced by the liver. However, less invasive and more reliable therapeutic approaches have been proposed for FAP therapy, namely based on drugs acting as inhibitors of amyloid formation or as amyloid disruptors. We have recently reported that epigallocatechin-3-gallate (EGCG), the most abundant catechin in green tea, is able to inhibit TTR aggregation and fibril formation, “in vitro” and in a cellular system, and is also able to disrupt pre-formed amyloid fibrils “in vitro”.

**Methodology and Principal Findings:**

In the present study, we assessed the effect of EGCG subchronic administration on TTR amyloidogenesis “in vivo”, using well characterized animal models for FAP. Semiquantitative immunohistochemistry (SQ-IHC) and Western blot analysis of mice tissues after treatment demonstrated that EGCG inhibits TTR toxic aggregates deposition in about 50% along the gastrointestinal tract (GI) and peripheral nervous system (PNS). Moreover EGCG treatment considerably lowered levels of several biomarkers associated with non-fibrillar TTR deposition, namely endoplasmic reticulum (ER)-stress, protein oxidation and apoptosis markers. Treatment of old FAP mice with EGCG resulted not only in the decrease of non-fibrillar TTR deposition but also in disaggregation of amyloid deposits. Consistently, matrix metalloproteinase (MMP)-9 and serum amyloid P component (SAP), both markers of amyloid deposition, were also found reduced in treated old FAP mice.

**Conclusions and Significance:**

The dual effect of EGCG both as TTR aggregation inhibitor and amyloid fibril disruptor together with the high tolerability and low toxicity of EGCG in humans, point towards the potential use of this compound, or optimized derivatives, in the treatment of TTR-related amyloidoses.

## Introduction

Familial amyloidotic polyneuropathy (FAP) is an autosomal dominant hereditary disease characterized by the extracellular deposition of nonbranching cross β-pleated sheet fibrils in the connective tissue, with a special involvement of the peripheral nervous system (PNS), both autonomic and motor. The onset of clinical symptoms generally occurs before age 40 with fast progression to death within 10 to 20 years. Amyloid deposits are mainly composed by transthyretin (TTR), being methionine for valine substitution at position 30, TTR V30M, the most common molecular abnormality in TTR [1]. More than one hundred amyloidogenic mutations have been reported, presenting some clinical heterogeneity but typically with peripheral nervous system (PNS) involvement [2].

Although the precise molecular mechanisms underlying TTR fibrillogenesis have not been yet fully disclosed, it is widely accepted that this process involves TTR tetramer dissociation, leading to partially unfolded monomers which self-assemble originating non-fibrillar aggregates, protofibrils and mature amyloid fibrils [3], [4]. Different TTR amyloidogenic species have been observed “in vivo” as the analysis of nerve biopsy samples from asymptomatic patients showed small non-fibrillar TTR aggregates that in later stages of the disease coexist with amyloid deposits [5]. Moreover, cytotoxicity of early TTR prefibrillar aggregates, and not mature fibrils, was demonstrated by their ability to induce oxidative and inflammatory stress in neuronal cells, ultimately leading to cell dysfunction and death [5].

So far, the only treatment of proven efficacy for FAP is orthotopic liver transplantation, in which the main source of mutant TTR is eliminated. This therapeutic approach is extremely invasive and not all patients benefit from clinical improvement [6]. Hence, less aggressive therapies for the treatment of TTR amyloidosis such as stabilization of the soluble circulating amyloid precursor, inhibition of aggregation of amyloidogenic intermediates and disruption of insoluble deposits have been proposed [7]. Recently, our group reported that epigallocatechin-3-gallate (EGCG), the main polyphenolic constituent of green tea, binds to soluble TTR, increases protein resistance to dissociation, and inhibits TTR fibril formation “in vitro” [8], [9]. Unexpectedly, EGCG-TTR interaction does not encompass the thyroxine (T_4_, a physiological TTR ligand) binding pocket [8], [10], which has been the primary target for structure-based design of TTR kinetic stabilizers [11]. Instead, EGCG binds at the surface of the protein in a region involving amino acid residues at the interface of both dimers in the molecule promoting tetramer conformational stabilization [10]. Furthermore, it has been shown that EGCG disrupts mature TTR preformed fibrils “in vitro” [8], [9]. Thus, EGCG acts not only as inhibitor of TTR amyloid formation but also as disruptor of amyloid fibrils.

Based on these findings, we reasoned that EGCG might impair TTR amyloid fibril formation and its associated cytotoxicity “in vivo”. Therefore, we decided to test the effect of subchronic administration of EGCG “in vivo”, using two distinct, well established, animal models: hTTR V30M mice [12] and hTTR V30M/HSF mice [13]. In transgenic hTTR V30M mice non-fibrillar deposition of mutant TTR starts at six months of age, with particular involvement of the gastrointestinal tract (GI). Initial aggregates evolve into mature amyloid deposits normally when mice are over 12 months of age [14]. Although this mice model offers a useful tool for the study of FAP pathology and treatment, it does not recapitulate whole human disease features, since the PNS is spared. Hence, a different animal model comparable to hTTR V30M mice but lacking the heat shock factor 1 (HSF1) has been generated, the TTR V30M/HSF mice [13]. This animal model resembles human pathology by presenting TTR deposition in PNS. We herein report the results of EGCG administration “in vivo” using the above referred FAP transgenic mice models.

## Materials and Methods

### Ethics statement

All animal experiments performed were approved by the Portuguese General Veterinarian Board (authorization number 024976 from DGV-Portugal) and animals were kept and used strictly in accordance with National rules and the European Communities Council Directive (86/609/EEC), for the care and handling of laboratory animals.

### Reagents

EGCG was purchased from Cayman Chemicals (Ann Arbor, USA). Histoclear was purchased from National Diagnostics (Atlanta, GA, USA). Rabbit polyclonal anti-TTR was from Dako (Carpinteria, CA, USA). Goat polyclonal anti-BiP and rabbit polyclonal anti-Fas were from Santa Cruz (CA, USA). Rabbit polyclonal anti-3-nitrotyrosine was from Chemicon (Temecula, CA, USA). Rabbit polyclonal anti-matrix metalloproteinase (MMP)-9 and rabbit polyclonal anti-BiP were from Abcam (Cambridge, UK). Rabbit polyclonal anti-P-eIF2α was from Biosource (CA, USA). Mouse monoclonal anti-actin was from Sigma (St. Louis, MO, USA). Sheep polyclonal anti-serum amyloid P component (SAP) was a kind gift from the laboratory of Professor Pepys, Royal Free Hospital, London. The biotin-extravidin peroxidase kit was from Sigma. Bradford protein assay was from BioRad (CA, USA). Proteases inhibitors, Hybond-C membranes and ECL® (enhanced chemiluminescence) were from GE Healthcare.

### Transgenic mice

Transgenic mice for human TTR V30M in a TTR null background [12], labeled as hTTR V30M mice 4.5 months-old (n = 10) or 17 months-old (n = 11) were treated with EGCG administrated in the drinking water (100 mg/kg/day) over 6 weeks. The EGCG solution was freshly prepared every day and protected from light. Age matched control animals were maintained in the same conditions and given water alone (n = 6 and n = 8, respectively). An additional experiment used 4.5 months-old mice expressing human TTR V30M in a TTR null background heterozygous for the heat shock transcription factor 1 (HSF1) [13] labeled hTTR V30M/HSF mice. Similarly, mice (n = 8) were treated for the same period of time with similar EGCG dosage. Age matched mice receiving water alone were used as controls (n = 5). After the treatment period, animals were sacrificed following anesthesia with ketamine/xylazine. Mice tissues in particular whole gastrointestinal tract (GI), including esophagus, stomach, colon and duodenum, as well as the dorsal root ganglia an sciatic nerve in the case of the hTTR TTR V30M/HSF mice, were immediately excised and frozen at −70°C or fixed in 4% neutral buffered formalin and embedded in paraffin for light microscopy techniques.

### Isoelectric focusing (IEF) in semi-denaturing conditions

Thirty microliters of plasma from EGCG treated mice and from controls (non-treated mice) were subjected to native electrophoresis (PAGE). The TTR gel band was excised and applied to a semi-denaturing (4 M urea) pH 4–6.5 gradient isoelectric focusing (IEF) gel run for 6 h at 1200 V [15]. Proteins were stained with Coomassie Blue. The gels were scanned and subjected to densitometry analysis using the ImageQuant program.

### Immunohistochemistry

Tissue sections (5 µm thick) were deparaffinated in histoclear and dehydrated in a descent alcohol series. Endogenous peroxidase activity was inhibited with 3% hydrogen peroxide/100% methanol, and sections were blocked in 4% fetal bovine serum and 1% bovine serum albumin in PBS. The primary antibodies and the respective dilutions used were: rabbit polyclonal anti-TTR (1∶1000), goat polyclonal anti-BiP (1∶50), rabbit polyclonal anti-Fas (1∶200), rabbit polyclonal anti-3-nitrotyrosine (1∶500), rabbit polyclonal anti-matrix metalloproteinase (MMP)-9 (1∶1500), sheep polyclonal anti-serum amyloid P component (SAP) (1∶2000), which were diluted in blocking solution and incubated overnight at 4°C. Antigen visualization was performed with the biotin-extravidin peroxidase kit using hydrogen peroxide and diaminobenzidine as substrate and chromogen, respectively. Immunohistochemistry analysis was carried out independently by two investigators unaware of the origin of the tested tissue sections. Semi-quantitative immunohistochemical (SQ-IHC) analysis was performed using Scion Image software. This application enables the measurement of the area occupied by pixels corresponding to the immunohistochemical substrate's color that is normalized relatively to the total area. Each slide was analyzed in five different representative areas.

### Congo Red staining

The presence of amyloid deposits in tissue sections was investigated by Congo Red (CR) staining and observation under polarized light. Briefly, deparaffinated tissues sections were incubated for 20 min with 0.01% NaOH in 80% ethanol saturated with NaCl followed by staining with 0.5% Congo Red in the previous solution [16]. Following the preparations were washed with water, stained with hematoxilin and analyzed under polarized light. Amyloid was identified by distinctive green birefringence.

### Total protein extracts and Western blot analysis

Mice tissues (approximately 5 mg) in particular esophagus, stomach, colon and dorsal root ganglia were homogenized on ice in a small glass rod homogenizer in 1 mL of lysis buffer containing 5 mM EDTA, 2 mM EGTA, 20 mM MOPS, 1% Triton X-100, 1 mM PMSF and a Protease Inhibitor Mix (GE Healthcare). After centrifugation (14,000 rpm for 30 min at 4°C) protein concentration in the supernatant was determined by the Bradford protein assay.

Fifty µg of total protein from each tissue were separated by 15% SDS-PAGE and transferred onto a nitrocellulose Hybond-C membrane using a Mini Trans-Blot Cell (BioRad) system. The primary antibodies and the respective dilutions used were: rabbit polyclonal anti-TTR (1∶1000), rabbit polyclonal anti-BiP (1∶1000), rabbit polyclonal anti-P-eIF2α (1∶500) and mouse monoclonal anti-actin (1∶5000). Detection was performed with ECL® (enhanced chemiluminescence). Quantification of TTR, BiP or P-eIF2α levels was performed by densitometry using the ImageQuant 5.1 software (Molecular Dynamics). Density values were normalized with actin expression. Results are presented as normalized density ± SD.

### Quantification of TTR levels in mice plasma

Mice plasma TTR was quantified by Rocket Electroimmunodiffusion. In brief, plasma samples were diluted at a ratio of 1∶10 and applied into an agarose gel containing rabbit polyclonal anti-TTR antibody (1∶150). After electrophoresis, rocket-like precipitates of antigen-antibody complexes were formed along the axis of migration being the length proportional to antigen concentration.

### Statistical analyses

All data examined were expressed as mean values ± standard deviation (SD). Comparison between groups was made using the Student's t-test. A *P*-value of less than 0.05 was considered statistically significant (*P<0.05; **P<0.01; ***P<0.005).

## Results

### EGCG decreases non-fibrillar TTR deposition and associated biomarkers

Based in previous “in vitro” studies indicating that EGCG inhibits TTR amyloid fibril formation [8], [9], [10] we decided to test its effect “in vivo” using FAP mice models. We started by treating 4.5 months-old hTTR V30M mice, thus before TTR tissue deposition [12]. EGCG was administrated orally in the drinking water at a concentration of 100 mg/kg/day for 6 weeks period. This treatment period was chosen according to results from pilot assays of 4 and 6 weeks treatment. EGCG dosage was selected based upon available “in vivo” reports [17], and also taking into account both its oral acute (LD50 = 2170 mg/kg) and subchronic toxicity [18].

As predicted, this dosage did not produce adverse side effects, as no significant difference was observed in body weight, behavior or mortality between animals treated with EGCG and age matched untreated controls. Quantification of TTR in mice plasma from untreated (n = 6) and from EGCG treated (n = 10) hTTR V30M mice revealed no significant difference between the two groups (356.0±79.9 µg TTR/mL and 374.6±38.5 µg TTR/mL, respectively), demonstrating that EGCG treatment did not interfere with TTR expression “in vivo” or with TTR plasma level.

To investigate the effect of EGCG treatment on TTR stability we analyzed serum samples from treated mice and from controls (non-treated) by isoelectric focusing (IEF) under semi-dissociating conditions [15]. The results revealed a two fold increase of the ratio of TTR tetramer over total TTR as calculated by densitometry analysis of Coomassie blue stained protein (Fig. S1) indicating an increase of serum TTR stability in vivo after treatment with EGCG.

**Figure 1:**
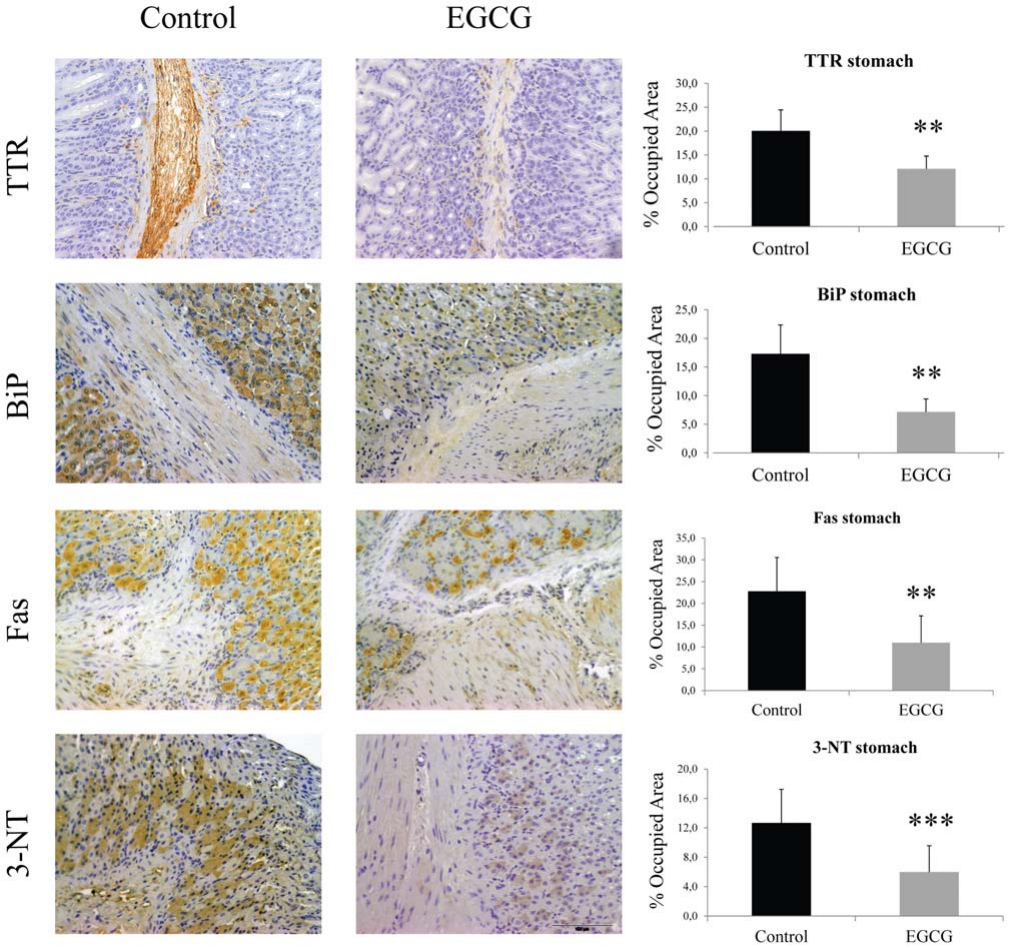
EGCG treatment decreases TTR deposition and associated biomarkers in stomach of hTTR V30M mice. Representative immunohistochemistry of TTR, BiP, Fas and 3-nitrotyrosine in stomach of hTTR V30M mice treated with EGCG (right panels; n = 10) and age-matched controls (left panels; n = 6). Scale bar 100 µm. Histogram: quantification of immunohistochemical images is represented as percentage of occupied area ± SD (**P<0.01; ***P<0.005).

**Figure 2:**
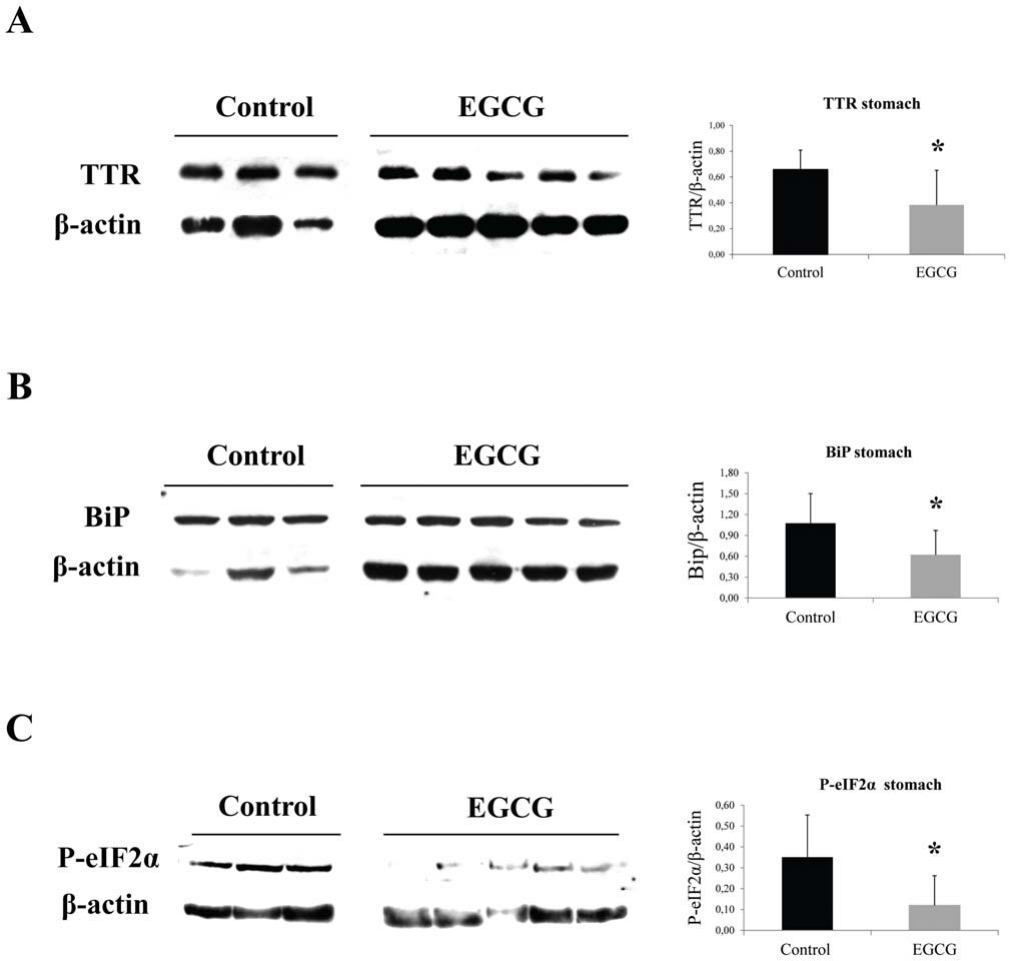
EGCG treatment decreases ER-stress markers associated with TTR deposition in stomach of hTTR V30M mice. Representative (**A**) anti-TTR, (**B**) anti-BiP and (**C**) anti-P-eIF2α Western blots of stomachs from TTR V30M mice treated with EGCG and non-treated mice. Histogram: normalized TTR/β-actin, BiP/β-actin and anti-P-eIF2α/β-actin density quantifications ±SD (*P<0.05).

**Figure 3:**
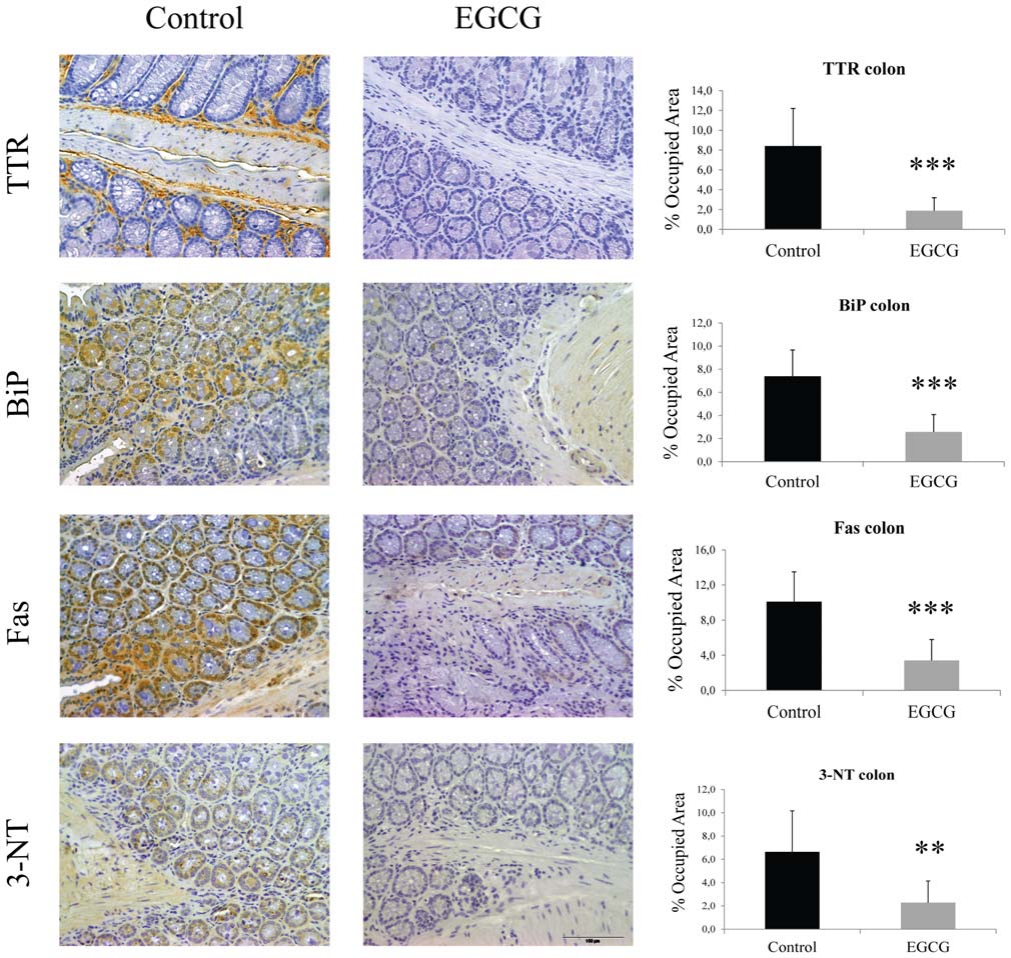
EGCG treatment decreases TTR deposition and associated biomarkers in colon of hTTR V30M mice. Representative immunohistochemistry of TTR, BiP, Fas and 3-nitrotyrosine in colon of hTTR V30M mice treated with EGCG (right panels; n = 10) and age-matched non-treated animals (left panels; n = 6). Scale bar 100 µm. Histogram: quantification of immunohistochemical images is represented as percentage of occupied area ± SD (**P<0.01; ***P<0.005).

We next assessed the effect of EGCG on non-fibrillar TTR deposition on different tissues by semi quantitative immunohistochemical analysis (SQ-IHC). At the end of the treatment, mice were 6 month-old and, as expected at this age, untreated mice revealed widespread TTR staining along the gastrointestinal (GI) tract. In contrast, EGCG treated mice presented significant reduction of TTR load in all GI tract organs analyzed. In stomach, the major organ of TTR deposition in hTTR V30M mice, we detected a decrease of approximately 40% of TTR aggregates as can be seen in the representative IHC images and respective quantification (**Fig. 1**, upper panels). These results were further corroborated by quantification of TTR in tissue lysates by Western blot (**Fig. 2A**). Moreover, the highest reduction in TTR staining was obtained in the intestine, particularly in colon (77% decrease) (**Fig. 3**, upper panels) and duodenum (Fig. S2).

Then we investigated several tissue markers formerly associated with TTR extracellular deposition [5], [19], [20]. Two of those are endoplasmic reticulum (ER)-stress markers, namely ER-resident chaperone BiP and the phosphorylated eukaryotic initiation factor 2 (P-eIF2α) typically upregulated in FAP patients [19]. The results showed that hTTR V30M mice treated with EGCG displayed significant decrease of BiP expression throughout the gut, including stomach (60% decrease) and colon (65% decrease), as can be seen in **Fig. 1** and **3**, respectively. In duodenum BiP expression was also significantly reduced (Fig. S2). BiP and P-eIF2α quantification by Western blot analysis of stomach lysates further corroborated the IHC results (**Fig. 2B and C**, respectively).

We also investigated the levels of the apoptotic biomarker death receptor Fas (CD95) usually increased in tissues of patients with TTR deposition [20]. Immunostaining analysis of mice tissues showed that treated mice presented reduced levels of Fas in the stomach, colon (**Fig. 1** and **3**, respectively) and duodenum (Fig. S2).

Tissue oxidative stress was assessed by evaluation of 3-nitrotyrosine (3-NT), a marker for protein peroxynitrite-mediated nitration. hTTR V30M treated mice revealed significant decrease of 3-NT immunostaining principally in colon (**Fig. 3**, bottom panels) and duodenum (Fig. S2); 3-NT was also decreased in the stomach (**Fig. 1**, bottom panels).

To test the effect of EGCG on TTR deposition in the PNS we performed a similar study with hTTR V30M/HSF mice using the same EGCG dosage and time of treatment. In accordance with the results for hTTR V30M mice, plasma TTR levels from hTTR V30M/HSF control mice (n = 5; 363.9±65.7 µg TTR/mL) did not differ significantly from the levels of EGCG treated animals (n = 8; 348.6±67.5 µg TTR/mL). SQ-IHC analysis of tissue sections showed that EGCG treatment significantly lowered TTR aggregates deposition in the GI tract, namely in stomach and colon (Fig. S3A and S3B, respectively). Accordingly, a significant reduction in BiP levels was observed along the digestive tract, particularly in stomach and colon (Fig. S3A and S3B). These results are in conformity with the previous data obtained for the hTTR V30M mice. Western blot analysis against BiP of tissue lysates further validated the IHC data (Fig. S4A).

**Figure 4:**
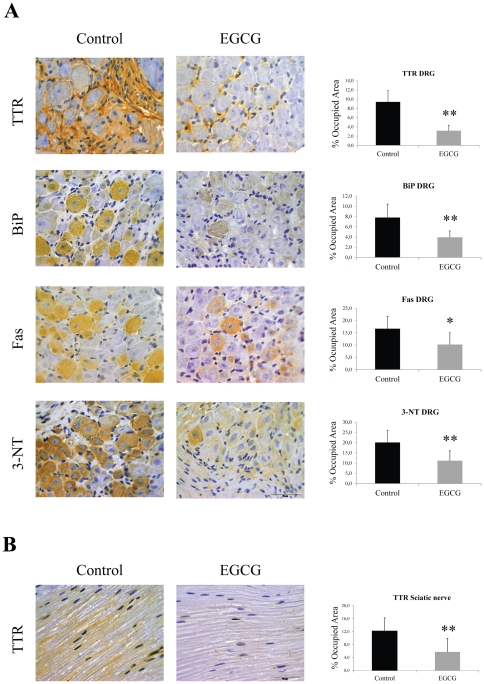
EGCG treatment decreases TTR deposition and associated biomarkers in the peripheral nervous system (PNS) of hTTR V30M/HSF mice. (**A**) Representative immunohistochemistry of TTR, BiP, Fas and 3-nitrotyrosine in dorsal root ganglia of hTTR V30M/HSF treated with EGCG for 6 weeks (right panels; n = 8) and age-matched controls (left panels; n = 5). Scale bar 50 µm. (**B**) Immunohistochemistry of TTR in sciatic nerve of hTTR V30M treated mice (right panels; n = 8) and age-matched non-treated animals (left panels; n = 5). Scale bar 50 µm. Histograms: quantification of the levels of the referred markers expressed as percentage of occupied area ± SD (*P<0.05; **P<0.01).

Concerning the involvement of PNS, hTTR V30M/HSF mice treated with EGCG had significant reduction of extracellular TTR deposition in dorsal root ganglia (DRG) (66% decrease) as exemplified in representative IHC pictures and in the histograms (**Fig. 4A**, upper panels). A comparable effect on TTR deposition was observed in the sciatic nerve of EGCG treated animals (**Fig. 4B**). ER-stress activation was also assessed in PNS, and the results showed that EGCG treated hTTR V30M/HSF mice presented approximately 50% less of BiP than controls in DRG, as evident by IHC (**Fig. 4A**) and Western blot (Fig. S4B). Moreover, Fas expression and 3-NT intracellular level were found considerably lowered not only in DRG from EGCG treated mice (**Fig. 4A**) but also in the sciatic nerve (data not shown). These results sustained that EGCG interferes with TTR deposition in both GI tract and PNS.

### EGCG disrupts amyloid deposits “in vivo”

Previous “in vitro” results revealed that EGCG is able to disrupt preformed amyloid fibrils [8], [9]. This prompted us to investigate the effect of EGCG in aged hTTR V30M mice (17 months-old) presenting amyloid deposits.

**Figure 5:**
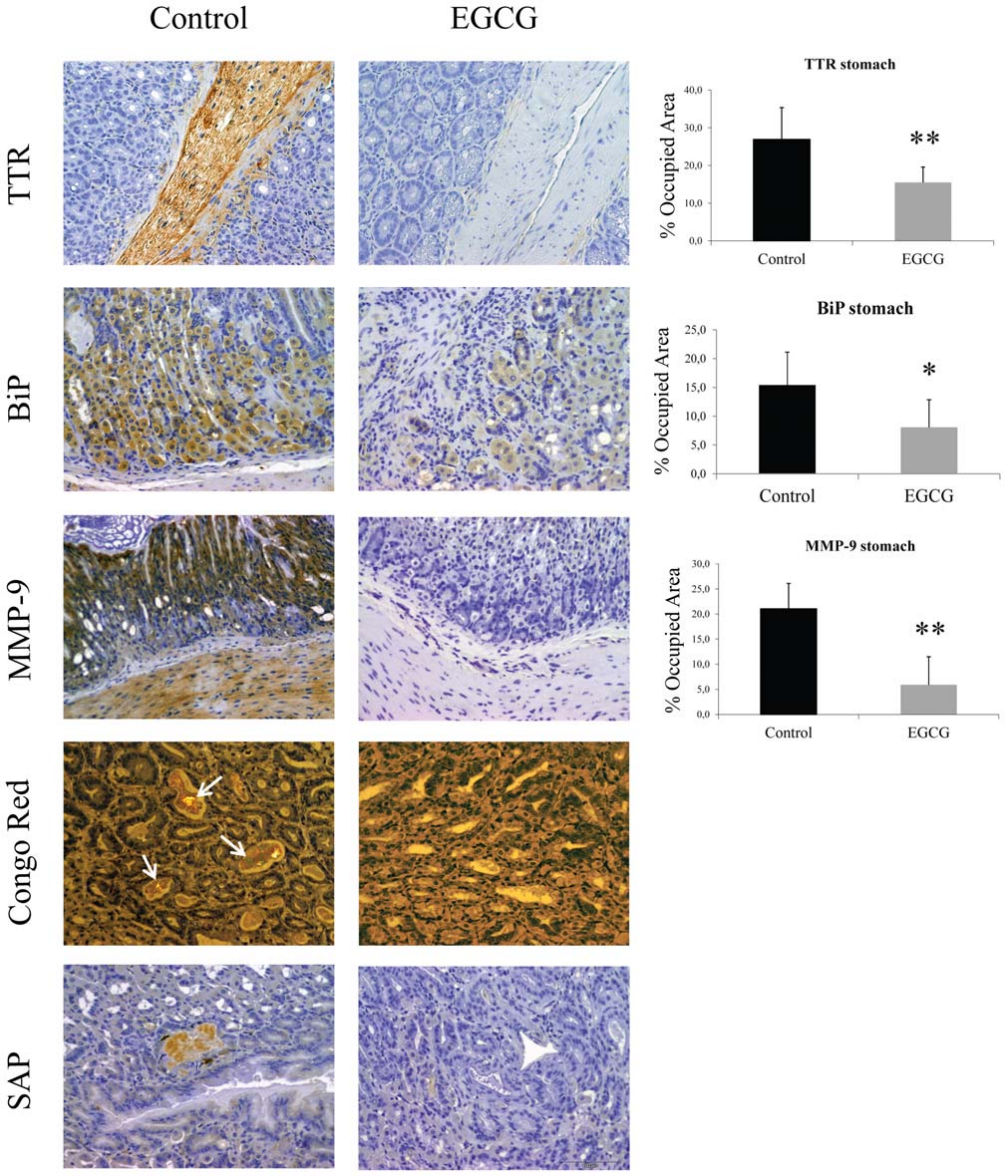
EGCG treatment decreases TTR deposition and associated biomarkers in the gastrointestinal tract of old hTTR V30M mice. Immunohistochemistry and Congo Red analysis of stomachs from aged hTTR V30M mice treated with EGCG (right panels; n = 11) and aged-matched controls (left panels; n = 8). The top 3 panels represent TTR, BiP and MMP-9 immunohistochemistry. The bottom two panels are representative Congo Red staining (white arrows pointing at CR birefringence in a non-treated mouse) and mouse SAP immunohistochemistry. Scale bar 100 µm. Histograms: quantification of the levels of the referred markers expressed as percentage of occupied area ± SD (*P<0.05; **P<0.01).

Analysis of TTR deposition indicated that, as expected, control animals had higher levels of TTR deposition in all GI organs analyzed as compared with untreated younger hTTR V30M mice. Still, EGCG administration significantly reduced TTR load in aged mice, particularly in stomach (42% decrease) (**Fig. 5**), and colon (55% decrease) (data not shown).

**Figure 6:**
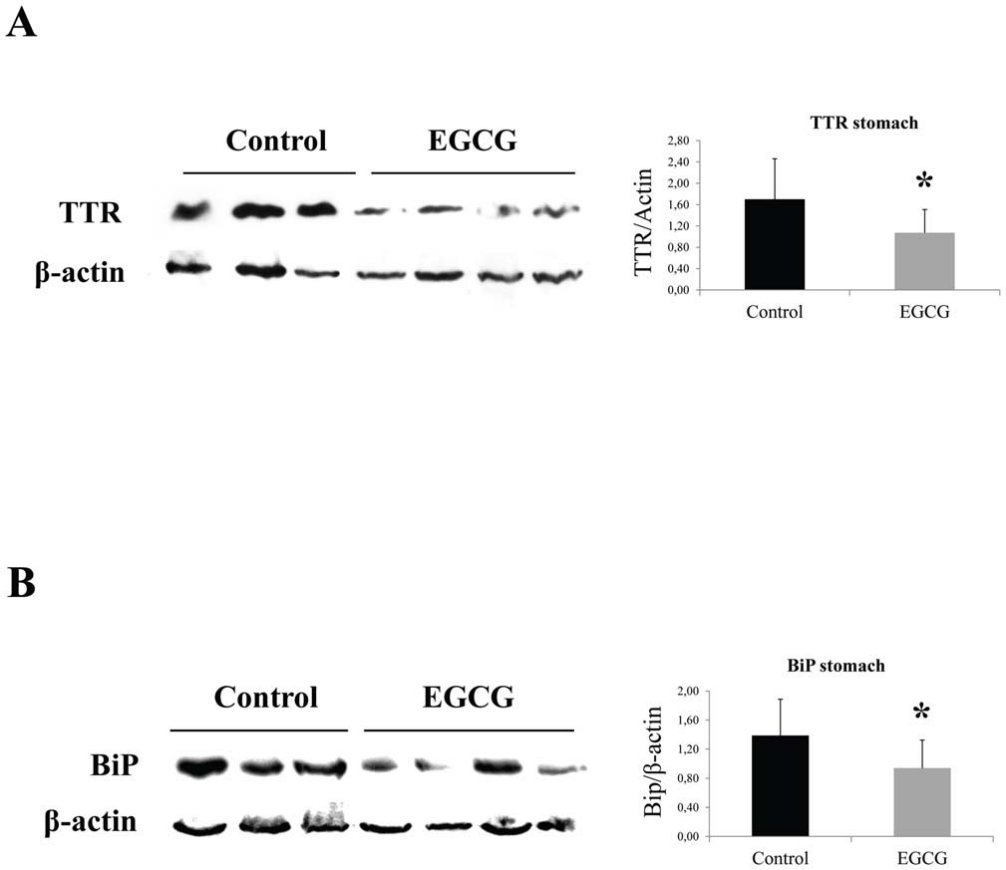
EGCG treatment decreases TTR amyloid deposition in the gastrointestinal tract of old hTTR V30M mice. Representative (**A**) anti-TTR and (**B**) anti-BiP Western blots of stomachs from aged hTTR V30M mice treated with EGCG and non-treated mice. Histogram: normalized TTR/β-actin and BiP/β-actin density quantifications ±SD (*P<0.05).

The effect of EGCG on the biomarkers tested above for young hTTR V30M mice were also assessed in aged mice. BiP was found significantly lowered in the stomach (approximately 50% decrease) of treated mice when comparing with untreated animals, as depicted by representative IHC in **Fig. 5**. The IHC results were further corroborated by Western blot analysis against BiP in stomach lysates (**Fig. 6B**). Accordingly, Fas levels were considerably decreased in EGCG treated animals, particularly in stomach and colon. Moreover, 3-NT was also found lowered in treated mice along the GI tract, specifically in stomach and colon (data not shown).

In the hTTR V30M strain used, mature amyloid fibril deposition typically begins when mice are over one year of age [14]; thus, in 17 month-old animals (at the beginning of treatment) TTR detection by IHC refers to whole deposited TTR, both aggregated and fibrillar forms. The mature amyloid material was detected by Congo Red (CR) staining in stomach sections, the primary target-organ in this particular mice model. The results showed that 5 out of 8 (63%) untreated animals presented fibrillar CR-positive material in stomach, whereas only 3 out of the 11 (27%) EGCG treated animals displayed CR green birefringence under polarized light (**Fig. 5**). Interestingly, the last referred animals (3 out of 11), apparently non- responder mice, showed significantly higher levels of SAP or MMP-9 (both markers for mature amyloid deposition) as compared to the corresponding group final average. These results indicate that EGCG affects TTR congophilic deposits “in vivo”, most probably through disaggregation.

TTR amyloid deposits are composed not only by the mutant protein, but also by other constituents such as serum amyloid P component (SAP) which are non-covalently associated with amyloid fibrils, protecting the amyloid peptide from proteolytic breakdown [21]. We assessed the presence of mouse SAP in the stomach from EGCG treated and untreated animals. Immunohistochemistry results indicated that only animals presenting TTR congophilic deposits (+/+), showed significant levels of SAP, while mice with non-fibrillar (+/−) and without (−/−) deposition presented lower or negligible staining. Since matrix metalloproteinase (MMP)-9 has been found upregulated in amyloid laden tissues in FAP [22] we also evaluated MMP-9 levels. SQ-IHC analysis revealed that MMP-9 was notably reduced (75% reduced) in EGCG treated animals as compared to untreated mice (**Fig. 5**).

## Discussion

There are multiple lines of compelling evidence indicating that EGCG can be useful for the prevention and treatment of a variety of protein misfolding diseases. Recent “in vitro” studies have shown that EGCG binds to natively unfolded proteins, such as α-synuclein (αS) and amyloid beta (Aβ), and redirects polypeptides from their standard self-assembly cascades into small off-pathway aggregates that are non-toxic to mammalian cells [23]. Regarding TTR-related amyloidosis, it has also been demonstrated, “in vitro” and “ex vivo”, that EGCG binds to TTR and increases its tetramer conformational stability. In addition, EGCG inhibits TTR aggregation “in vitro” and in a cell culture system [8], [9].

These findings, together with the pharmacokinetics [24] and favorable toxicological profile in mice [18], encouraged us to investigate the effect of subchronic administration of EGCG “in vivo”, using well established FAP mice models at different stages of TTR deposition. Data available on the pharmacokinetics indicated that a single dose intra-gastric administration of EGCG (75 mg/Kg) originates a peak plasma level of EGCG of 0.28±0.08 µmol/L and that when this administration was repeated after 6 h interval the levels of EGCG in blood were increased about 5.9 times and in tissues the levels were even higher [24], [25] . Since we used a superior dose of EGCG (100 mg/Kg) and a chronic administration, we expected that the levels of EGCG in plasma reached a plateau of concentration sufficient to substantially stabilize plasma TTR as we really found by IEF analysis of plasma TTR after mice treatment.

Most important, in this study, we demonstrate that EGCG lowers TTR systemic deposition in FAP mice, particularly in the gastrointestinal tract and peripheral nervous system. Consequently, associated biomarkers such as BiP, P-eIF2α, Fas and 3-nitrotyrosine were also reduced along the gastrointestinal tract of hTTR V30M and hTTR V30M/HSF and dorsal root ganglia of hTTR V30M/HSF mice.

As pointed out by several authors, the EGCG inhibitory effect on the amyloid formation cascade is not limited to early intermediates of fibrillogenesis, since EGCG is also able to efficiently remodel mature fibrils made from a variety of amyloidogenic proteins into smaller, non-toxic unstructured protein aggregates [8], [9], [26].

We also observed that EGCG displays amyloid disruptor activity “in vivo” in treated aged hTTR V30M mice, that simultaneously present non-fibrillar and fibrillar TTR forms, and found that EGCG treatment not only lowered systemic TTR load and associated biomarkers, but also disaggregated amyloid deposits as evidenced by Congo Red birefringence analysis of stomach sections (**Fig. 5**). It could be argued that TTR aggregates remodeling hide antibody reactivity, however TTR reduction under denaturing conditions in Western blots is also observed (**Fig. 6A**).

These results were further supported by substantial decrease of matrix metalloproteinase (MMP)-9 levels in EGCG treated mice, which was concomitant with amyloid clearance and indicative of inflammation reduction and matrix recovery. Nevertheless, several reports indicate EGCG as an inhibitor of MMP-9 expression [27], thus we cannot fully determine whether the significant MMP-9 reduction in treated animals was only due to EGCG-mediated TTR amyloid disaggregation or, in some extension, by direct action of EGCG on MMP-9 transcriptional activity. However, since serum amyloid P component (SAP) is a universal marker of amyloid its reduction points towards a direct effect of EGCG on TTR amyloid removal.

Though liver transplantation is the most efficient therapy available for FAP many patients present progression of the disease after hepatic transplantation and the procedure is not suitable for the treatment of a large number of patients. Hence, alternative therapeutic approaches including small TTR stabilizers [11], [28], [29], fibril disruptors [30], modulators of aggregate-induced toxicity [31], [32] or combined drug therapy [33] have been proposed. However, adverse side effects such as heart, liver or gastrointestinal dysfunction are associated with the use of some of these drugs “in vivo”, a major disadvantage when considering experimental research in FAP patients.

EGCG is the most abundant catechin in green tea and it has been widely recognized has safe in humans [34]. In addition, a recent study [35] on the green tea flavan-3-ols pharmacokinetics in humans revealed that their calculated bioavailability was approximately 39% and that EGCG was the only unmetabolized compound and the highest in absolute concentration. These findings might support previous reports indicating clinical benefit from green tea consumption (1.5–2 L/daily, equivalent of 600–800 mg EGCG) in patients with amyloid light chain (AL) amyloidosis [36], [37]. Very recently, Mereles and Hunstein [38] described several factors that may contribute to the increase of bioavailability of EGCG such as the concomitant ingestion of vitamin C, fish oil and piperine that should be taken into account when considering EGCG oral administration. Hence, only upcoming studies on chronic EGCG dietary supplementation in humans will further elucidate its plasma pharmacokinetics and tissue distribution.

Although EGCG may be used in different stages of disease progression we postulate that its potential therapeutic administration should preferentially address early phases of FAP since: (i) tissue deposition of small toxic TTR aggregates occurs at asymptomatic stage [14] (ii) EGCG inhibitory effect on TTR aggregation and associated cytotoxicity was more pronounced in younger FAP mice (iii) multiple organ dysfunction caused by space-occupying amyloid deposits may be clinically difficult to reverse.

Most of the TTR-related amyloidosis does not affect the central nervous system (CNS) but there are few TTR mutations (about 10 variants) associated with TTR amyloid deposition in the leptomeninges [39]. Therefore, EGCG ability to cross the blood-brain barrier [24] might be particularly suitable for those cases. It is well proven that tea catechins, in particular EGCG, possess free radical scavenging properties and act as biological antioxidants [40]. Moreover, EGCG can chelate metal ions, such as iron (III), to produce inactive complexes preventing metal-induced lipid peroxidation [41]. Thus, we do not disregard that together with its ability to directly modulate TTR fibrillogenesis, EGCG antioxidant and iron chelating activities may potentiate a neuroprotective effect “in vivo”. Hence, we propose EGCG, or optimized derivatives, as drug candidates for the treatment of FAP and amyloid diseases in general, paving the way for future clinical trials.

## Supporting Information

Figure S1 **EGCG treatment increases plasma TTR resistance to dissociation.** (**A**) Plasmas from 6 month-old hTTR V30M mice treated with EGCG (n = 10) and non-treated mice (n = 6) were subjected to isoelectric focusing analysis (IEF) under semi-denaturing conditions (4 M urea). These conditions allow the visualization of different molecular species corresponding to TTR monomers, an oxidized form of the monomer and tetramers. (**B**) The histogram shows TTR tetramer/total TTR bands ratio obtained after densitometry analysis of IEF gels) (**P<0.01).
(TIF)

Figure S2 **EGCG treatment decreases TTR deposition and associated biomarkers in duodenum of hTTR V30M mice.** Representative immunohistochemical analysis of TTR, BiP, Fas and 3-NT in duodenum of hTTR V30M mice treated with EGCG (right panels; n = 10) and age-matched controls (left panels; n = 6). Scale bar 100 µm. Histogram: quantification of immunohistochemical images is represented as percentage of occupied area ± SD (**P<0.01; ***P<0.005).
(TIF)

Figure S3 **EGCG treatment decreases TTR deposition and ER-stress marker BiP in the gastrointestinal tract of hTTR V30M/HSF mice.** (**A**) Immunohistochemistry of TTR (upper panels) and BiP (lower panels) in stomach of hTTR V30M/HSF mice treated with EGCG (right panels; n = 8) and age-matched controls (left panels; n = 5). Scale bar 100 µm. (**B**) Immunohistochemistry of TTR (upper panels) and BiP (lower panels) in colon of hTTR V30M mice/HSF treated with EGCG (right panels; n = 8) and controls (left panels; n = 5). Scale bar 100 µm. Histograms: quantification of immunohistochemical images is represented as percentage of occupied area ± SD (**P<0.01; ***P<0.005).
(TIF)

Figure S4 **EGCG treatment decreases ER-stress marker BiP in stomach and dorsal root ganglia of hTTR V30M/HSF mice.** Anti-BiP Western blot analysis of protein extracts from (**A**) stomachs and (**B**) dorsal root ganglia of hTTR V30M/HSF mice treated with EGCG and non-treated mice. Histogram: normalized BiP/β-actin density quantifications ±SD (*P<0.05).
(TIF)
