# Supplementary figures and images for: Epigallocatechin-3-Gallate as a Potential Therapeutic Drug for TTR-Related Amyloidosis: “In Vivo” Evidence from FAP Mice Models
Source: PLoS One. 2012 Jan 10;7(1):e29933. doi: 10.1371/journal.pone.0029933 (PMC3254632; doi:10.1371/journal.pone.0029933)

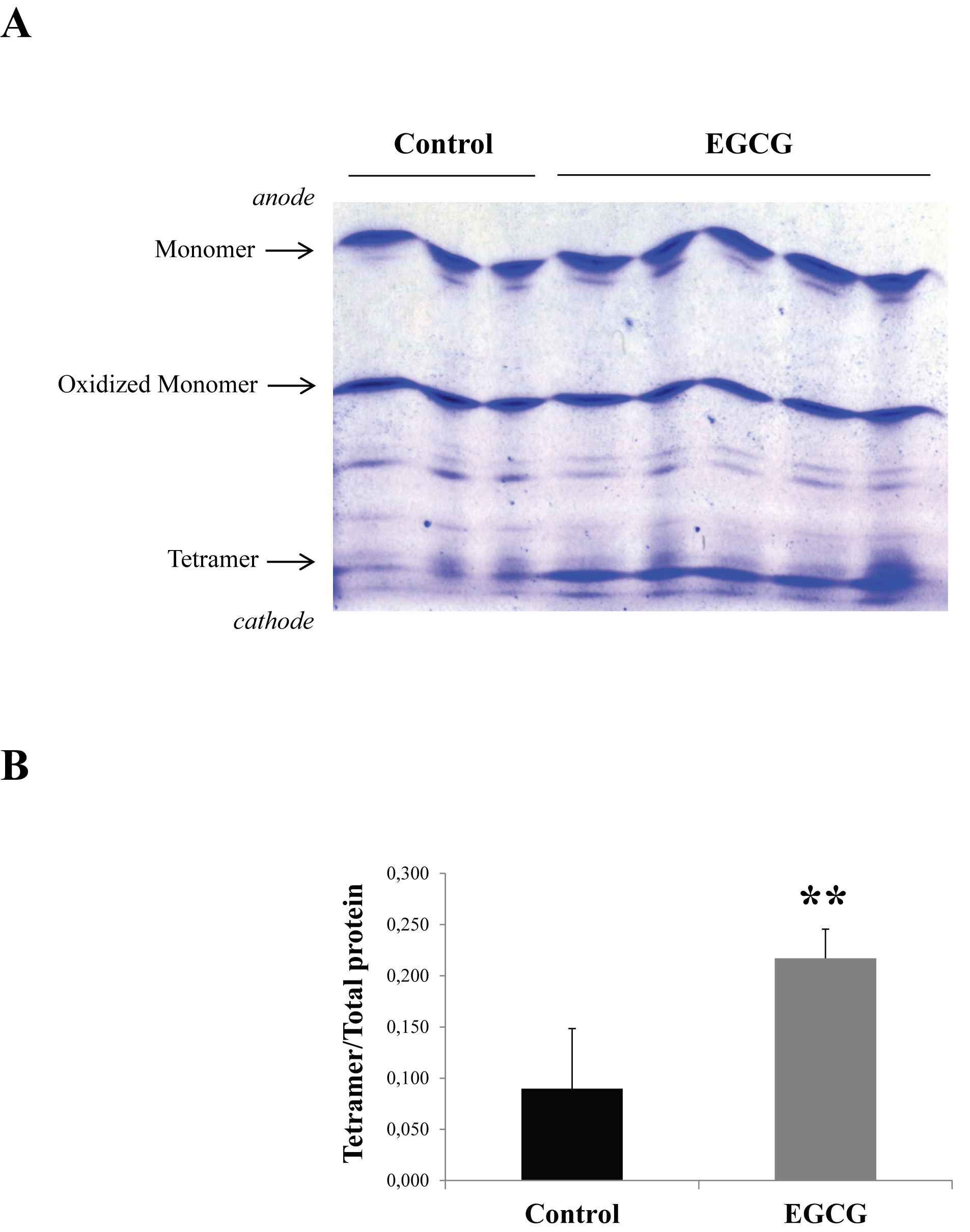

Supplement: Figure S1 — EGCG treatment increases plasma TTR resistance to dissociation. (A) Plasmas from 6 month-old hTTR V30M mice treated with EGCG (n = 10) and non-treated mice (n = 6) were subjected to isoelectric focusing analysis (IEF) under semi-denaturing conditions (4 M urea). These conditions allow the visualization of different molecular species corresponding to TTR monomers, an oxidized form of the monomer and tetramers. (B) The histogram shows TTR tetramer/total TTR bands ratio obtained after densitometry analysis of IEF gels) (**P<0.01). (TIF) [file pone.0029933.s001.tif]

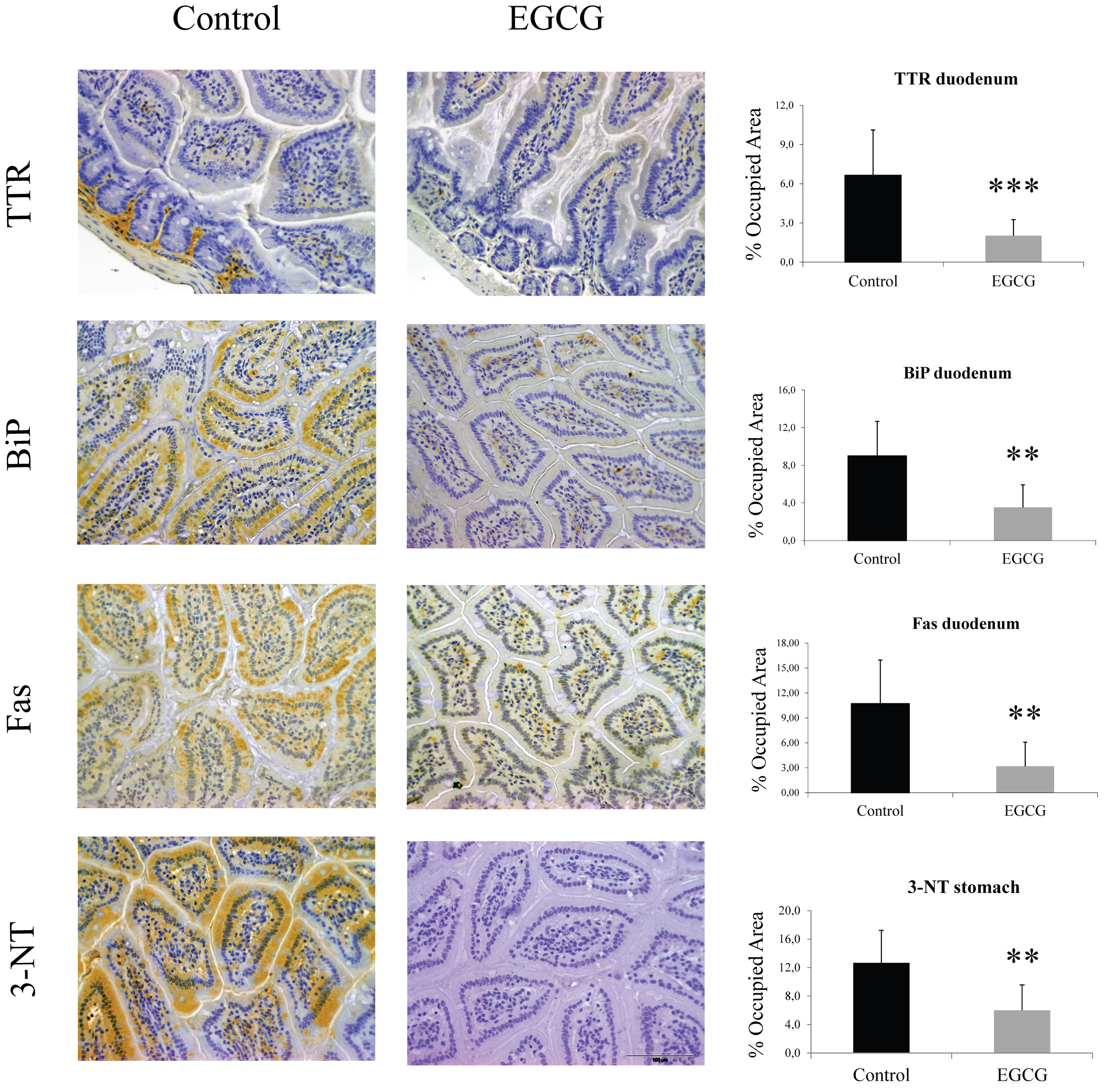

Supplement: Figure S2 — EGCG treatment decreases TTR deposition and associated biomarkers in duodenum of hTTR V30M mice. Representative immunohistochemical analysis of TTR, BiP, Fas and 3-NT in duodenum of hTTR V30M mice treated with EGCG (right panels; n = 10) and age-matched controls (left panels; n = 6). Scale bar 100 µm. Histogram: quantification of immunohistochemical images is represented as percentage of occupied area ± SD (**P<0.01; ***P<0.005). (TIF) [file pone.0029933.s002.tif]

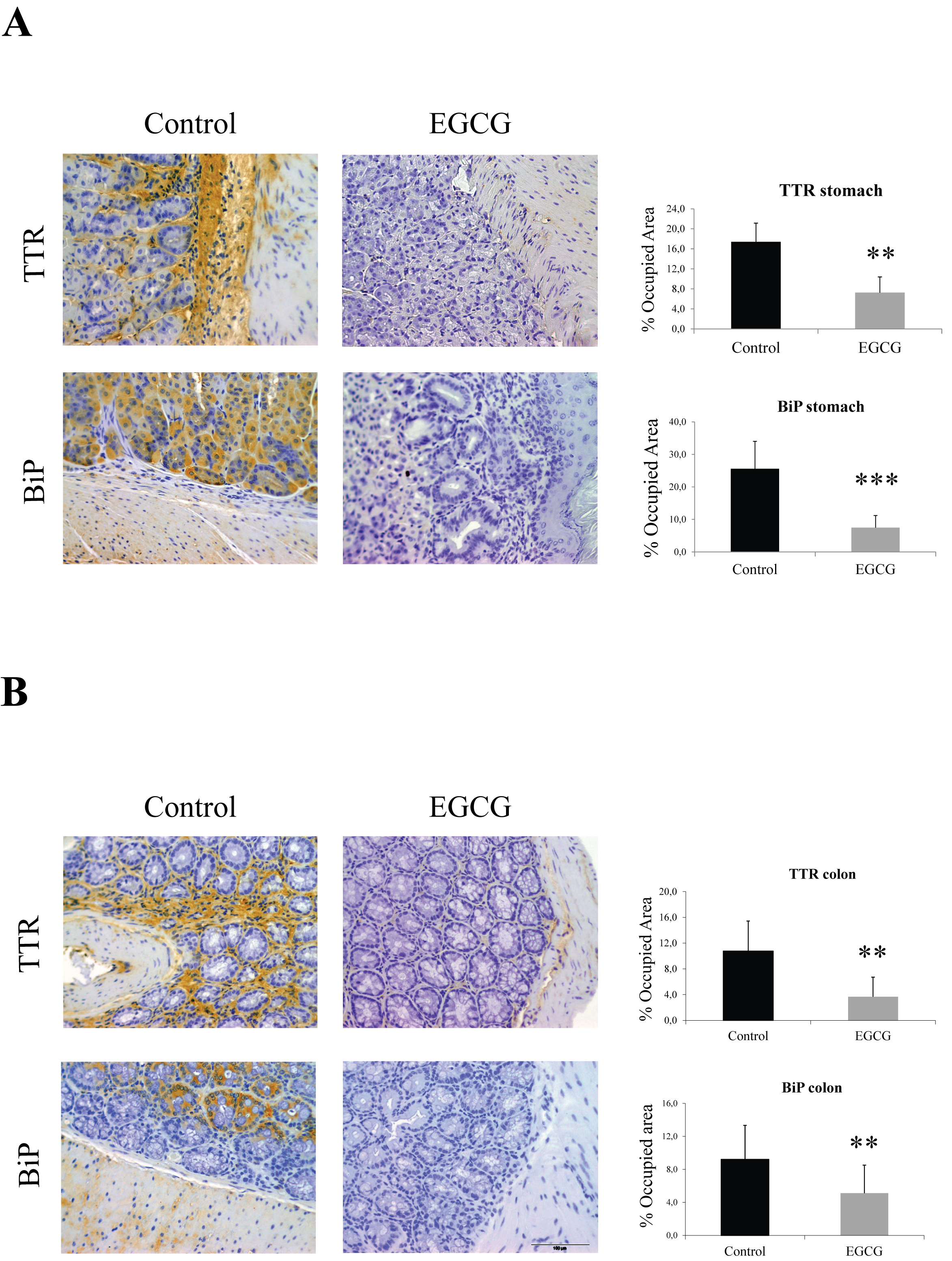

Supplement: Figure S3 — EGCG treatment decreases TTR deposition and ER-stress marker BiP in the gastrointestinal tract of hTTR V30M/HSF mice. (A) Immunohistochemistry of TTR (upper panels) and BiP (lower panels) in stomach of hTTR V30M/HSF mice treated with EGCG (right panels; n = 8) and age-matched controls (left panels; n = 5). Scale bar 100 µm. (B) Immunohistochemistry of TTR (upper panels) and BiP (lower panels) in colon of hTTR V30M mice/HSF treated with EGCG (right panels; n = 8) and controls (left panels; n = 5). Scale bar 100 µm. Histograms: quantification of immunohistochemical images is represented as percentage of occupied area ± SD (**P<0.01; ***P<0.005). (TIF) [file pone.0029933.s003.tif]

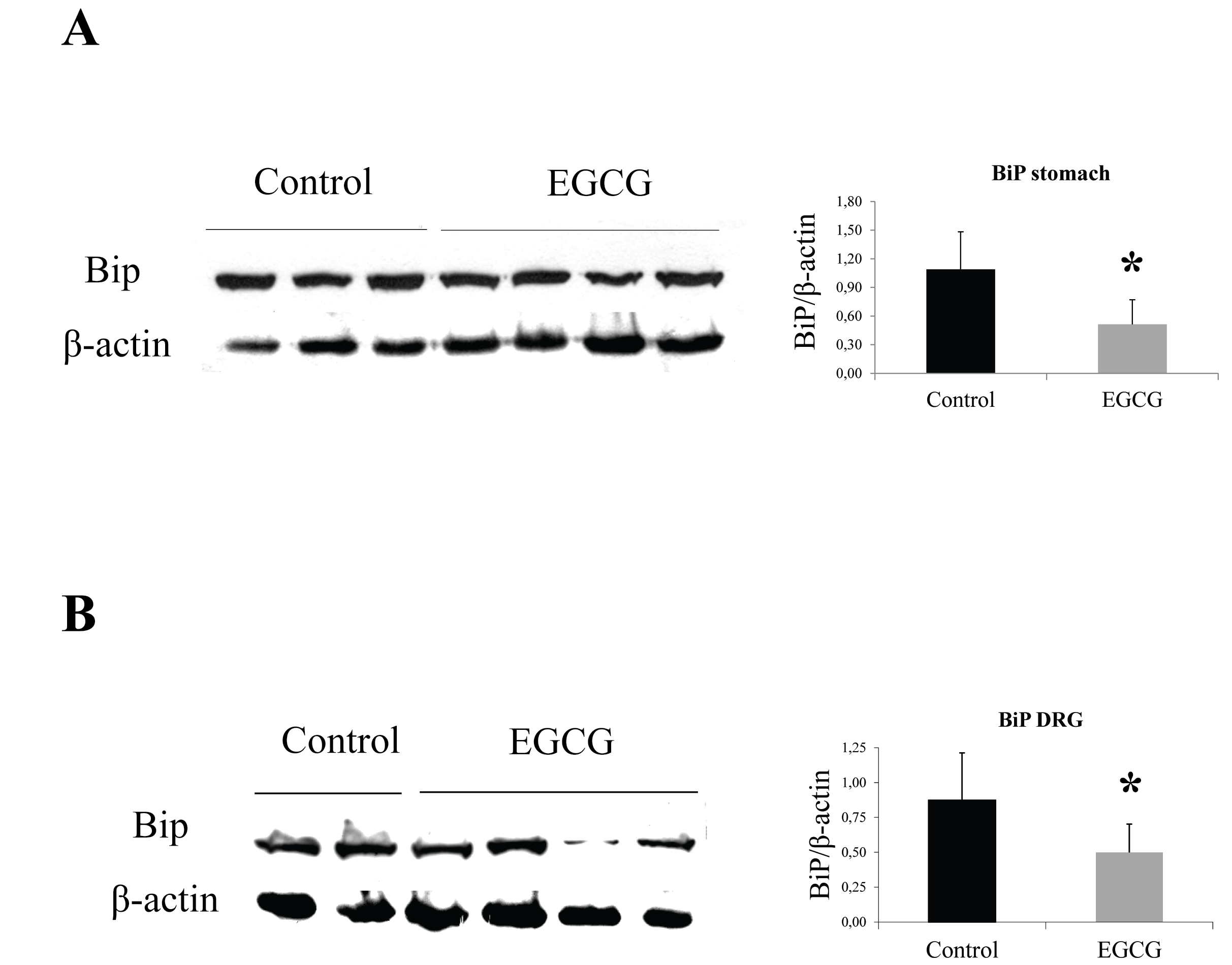

Supplement: Figure S4 — EGCG treatment decreases ER-stress marker BiP in stomach and dorsal root ganglia of hTTR V30M/HSF mice. Anti-BiP Western blot analysis of protein extracts from (A) stomachs and (B) dorsal root ganglia of hTTR V30M/HSF mice treated with EGCG and non-treated mice. Histogram: normalized BiP/β-actin density quantifications ±SD (*P<0.05). (TIF) [file pone.0029933.s004.tif]
